# Uptake of Nanotitania by Gingival Epithelial Cells Promotes Inflammatory Response and Is Accelerated by *Porphyromonas gingivalis* Lipopolysaccharide: An In Vitro Study

**DOI:** 10.3390/ijms22158084

**Published:** 2021-07-28

**Authors:** Shiho Sugawara, Taichi Ishikawa, Shu Sato, Hidemichi Kihara, Masayuki Taira, Minoru Sasaki, Hisatomo Kondo

**Affiliations:** 1 Department of Prosthodontics and Oral Implantology, School of Dentistry, Iwate Medical University, 1-3-27 Chuo-dori, Morioka 020-8505, Iwate, Japan; ssugawar@iwate-med.ac.jp (S.S.); hidemichi.khr@gmail.com (H.K.); hisakondo@gmail.com (H.K.); 2 Division of Molecular Microbiology, Department of Microbiology, Iwate Medical University, 1-1-1 Idai-dori, Morioka 028-3694, Iwate, Japan; msasaki@iwate-med.ac.jp; 3 Division of Dental Anesthesiology, Department of Reconstructive Oral and Maxillofacial Surgery, School of Dentistry, Iwate Medical University, 1-3-27 Chuo-dori, Morioka 020-8505, Iwate, Japan; shu2010sum41@outlook.com; 4 Department of Biomedical Engineering, Iwate Medical University, 1-1-1 Idai-dori, Morioka 028-3694, Iwate, Japan; mtaira@iwate-med.ac.jp

**Keywords:** dental implant, nanotitania, peri-implantitis, *Porphyromonas gingivalis*

## Abstract

Titanium is often used in the medical field and in dental implants due to its biocompatibility, but it has a high rate of leading to peri-implantitis, which progresses faster than periodontitis. Therefore, in the present study, the expression of cytokines from gingival epithelial cells by nanotitania was investigated, which is derived from titanium in the oral cavity, and the additional effect of *Porphyromonas* *gingivalis* (periodontopathic bacteria) lipopolysaccharide (PgLPS) was investigated. Ca9-22 cells were used as a gingival epithelial cell model and were cultured with nanotitania alone or with PgLPS. Cytokine expression was examined by reverse transcription-quantitative polymerase chain reaction and enzyme-linked immunosorbent assay. In addition, cellular uptake of nanotitania was observed in scanning electron microscopy images. The expression of interleukin (IL)-6 and IL-8 significantly increased in Ca9-22 cells by nanotitania treatment alone, and the expression was further increased by the presence of PgLPS. Nanotitania was observed to phagocytose Ca9-22 cells in a dose- and time-dependent manner. Furthermore, when the expression of IL-11, related to bone resorption, was investigated, a significant increase was confirmed by stimulation with nanotitania alone. Therefore, nanotitania could be associated with the onset and exacerbation of peri-implantitis, and the presence of periodontal pathogens may worsen the condition. Further clinical reports are needed to confirm these preliminary results.

## 1. Introduction

Dental implants provide structural, functional and aesthetic treatment, using artificial tooth roots (dental implants) instead of the original teeth that may be lost due to tooth decay, periodontal disease, trauma, tumors or a congenital reason. Accidental symptoms and complications in dental implant treatment include nerve damage, maxillary sinusitis, foreign matter insertion in the maxillary sinus, abnormal bleeding [1,2,3] and peri-implantitis. Though it is influenced by its definition, there is a report that the incidence of peri-implantitis is 28–56% [4,5]. These complications pose a major challenge in achieving the long-term success of implants. Natural teeth are maintained in the gingiva, cementum, periodontal ligament and alveolar bone, whereas dental implants are mainly maintained by osteointegration between the alveolar bone and the implant. Therefore, there is a difference in the structure of the tissue that maintains implants and natural teeth [6].

Once peri-implantitis develops, it progresses faster than periodontitis of natural teeth and involves a high degree of alveolar bone resorption [7]. Peri-implantitis, similar to periodontitis, is thought to be caused by the bacteria in dental plaque [8], but no firm consensus has been reached regarding its pathogenic mechanism or treatment.

Titanium forms a strong passivation oxide film (nanotitania) with a thickness of 4 nm that is not easily ionized, so it is not easily absorbed, has excellent durability and is often used as a prosthetic material [9,10]. However, the environment in the oral cavity where the dental implant is placed contains features such as saliva and occlusal forces that can impact the implant. Furthermore, the oral environment becomes strongly acidic in the presence of dental plaque, and neutrophils involved in inflammation release active oxygen [11]. While it is not clear if acidity or neutrophil infiltration is involved, in recent years, there have been increasing reports of titanium implant corrosion and discoloration [12] and titanium allergy [13] and these have also been reported to be related to peri-implantitis [14,15]. In addition, in the implant abutment joint, at the contact point between the titanium and the host cells, a physical and chemical reaction occurs, which may cause debris or elution-precipitates from the titanium material [16]. Titanium is thought to be stable in the oral cavity, but it is ionized by the chemical and physical reactions described above and can be converted into nano-sized titanium oxide (nanotitania) [17].

Recently, it has been reported that nanotitania can provoke an inflammatory response in macrophages to a similar degree as lipopolysaccharide from the periodontopathic bacteria *Porphyromonas gingivalis* (PgLPS) [18]. Peri-implantitis is thought to be mainly caused by oral bacteria, but it is possible that the action of nanotitania, which has not been thoroughly investigated, causes a reaction in immune cells and is involved in the destruction of periodontal tissue including the jawbone. In fact, it has been reported that nanotitania increases the expression of the interleukin (IL)-13α2 receptor in gingival epithelial cells and promotes the production of transforming growth factor β1 [19]. PgLPS can cause an inflammatory response in various types of cells, such as macrophages, fibroblasts, endothelial cells and gingival epithelial cells, through activation of Toll-like receptor 2 (TLR2) and TLR4 [20]. By the action of PgLPS, epithelial cells produce cytokines such as IL-1β, IL-6 and IL-8 [21,22]. As an inflammatory cytokine, IL-6 exhibits actions such as activation of vascular endothelial cells, activation of leukocytes and enhancement of leukocyte migration [23]. IL-8 is one of the major chemotactic factors (chemokines) for neutrophils [24,25], is the earliest mark of bacterial infections and is also a strong promoter of angiogenesis [26]. Although there are many reports investigating the effects of PgLPS in periodontal disease [27,28], few studies have reported on the impact of nanotitania particles that may appear in the process of implant treatment. Therefore, the present study investigated the effects of inflammatory cytokine expression in human gingival epithelial cell lines (Ca9-22) by both bacterial cellular components (PgLPS) and nanotitania, which are common in peri-implantitis and periodontitis.

## 2. Results

### 2.1. Nanotitania Upregulates the Expression of Inflammatory Cytokines

**Figure 1:**
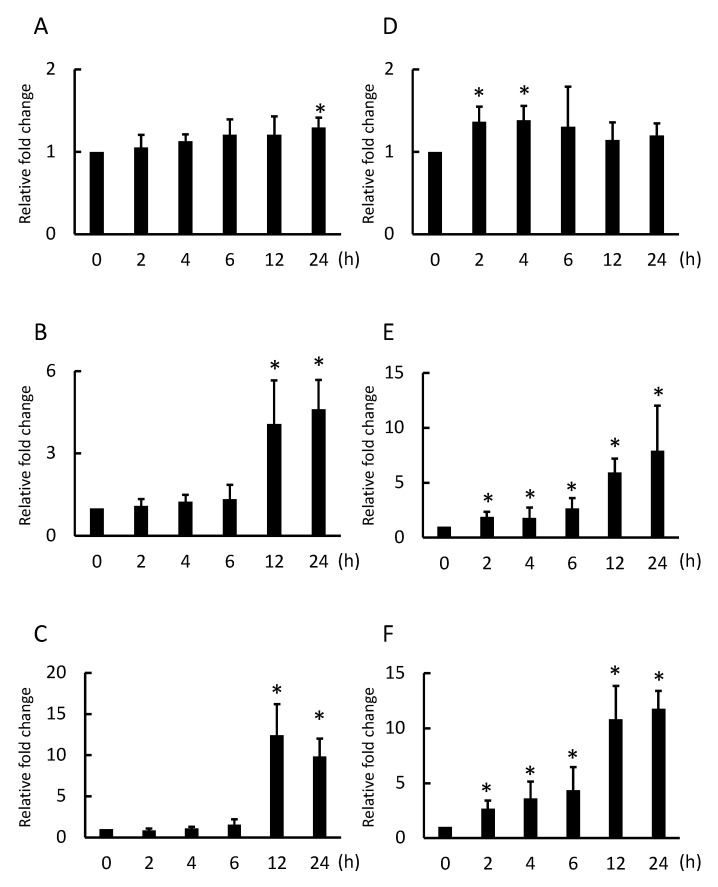
Expression levels of *IL-1β*, *IL-6* and *IL-8* mRNA were measured by quantitative polymerase chain reaction (qPCR), after incubation with 10 µg/mL nanotitania (**A**–**C**) or 100 µg/mL nanotitania (**D**–**F**). (**A**,**D**) *IL-1β*, (**B**,**E**) *IL-6* and (**C**,**F**) *IL-8* mRNA expression. Each data point indicates the average of six gene expression quantification results (n = 6). Bars indicate standard deviation, and asterisks indicate *p* < 0.05 compared with controls.

qPCR was performed to observe inflammatory cytokine expression. At 24 h incubation with 10 µg/mL nanotitania, *IL-1β* mRNA was slightly increased (Figure 1A). At 12 and 24 h incubation with 10 µg/mL nanotitania, *IL-6* mRNA was significantly increased (Figure 1B). Similar to *IL-6* mRNA expression, *IL-8* mRNA was significantly increased at 12 and 24 h incubation with 10 µg/mL nanotitania (Figure 1C). The peak at incubation with 100 µg/mL nanotitania tended to be faster and more intensified than that with 10 µg/mL nanotitania. *IL-1β* mRNA expression was increased at 2 and 4 h (Figure 1D) and *IL-6* and *IL-8* mRNA expression continued to significantly increase from 2 to 24 h incubation (Figure 1E,F).

### 2.2. Nanotitania Uptake by Gingival Epithelial Cells

**Figure 2:**
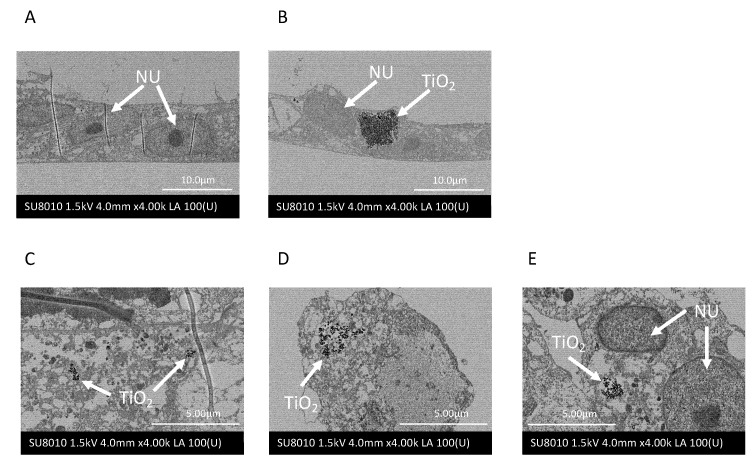
Scanning electron microscopy (SEM) images of Ca9-22 cells. (**A**) Control, (**B**) incubation with 100 µg/mL nanotitania for 1 h, (**C**) incubation with 10 µg/mL nanotitania for 1 h, (**D**) incubation with 10 µg/mL nanotitania for 12 h, (**E**) incubation with 10 µg/mL nanotitania for 24 h. NU, nuclear; TiO_2_, nanotitania.

SEM imaging was performed to observe whether nanotitania was incorporated into Ca9-22 cells. Control cells had no nanotitania (Figure 2A). Nanotitania uptake by Ca9-22 cells was observed when the cells were incubated at a concentration of 100 µg/mL for 1 h (Figure 2B). Incubated with 10 µg/mL nanotitania, a small amount of nanotitania uptake was observed at 1 h, and after 12 and 24 h incubation, the amount of uptake of nanotitania appeared to increase and the nanotitania appeared to aggregate (Figure 2C–E).

### 2.3. Endocytosis Inhibitor Blocks the Induction of Cytokine Production by the Uptake of Nanotitania in the Gingival Epithelium

**Figure 3:**
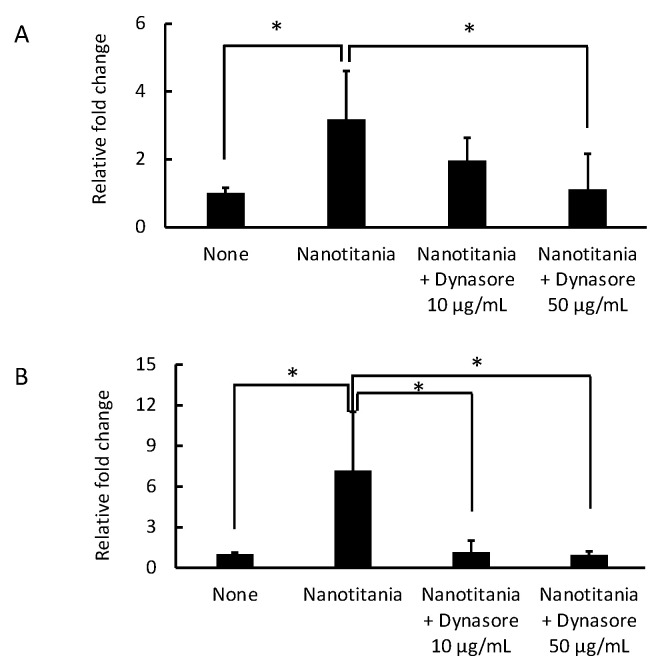
Effect of an endocytosis inhibitor on the expression of *IL-6* (**A**) and *IL-8* (**B**) mRNA expression was measured by qPCR. After the treatment with dynasore 10 or 50 mg/mL for 30 min, *IL-6* and *IL-8* mRNA expression was measured at 24 h incubation with 10 µg/mL nanotitania. Each data point indicates the average of six gene expression quantification results (n = 6). Bars indicate standard deviation, and asterisks indicate *p* < 0.05.

To confirm whether the induction of cytokine expression by nanotitania was due to the uptake of nanotitania, an endocytosis inhibitor (dynasore) was used. The induction of *IL-6* mRNA was significantly inhibited by 50 µg/mL dynasore (Figure 3A). Similarly, the induction of *IL-8* mRNA was significantly reduced by 50 µg/mL dynasore. In addition, the induction of *IL-8* mRNA was completely inhibited by 10 µg/mL as well as 50 µg/mL dynasore (Figure 3B).

### 2.4. Co-Culture with Nanotitania and PgLPS Upregulates the Expression of Inflammatory Cytokines

**Figure 4:**
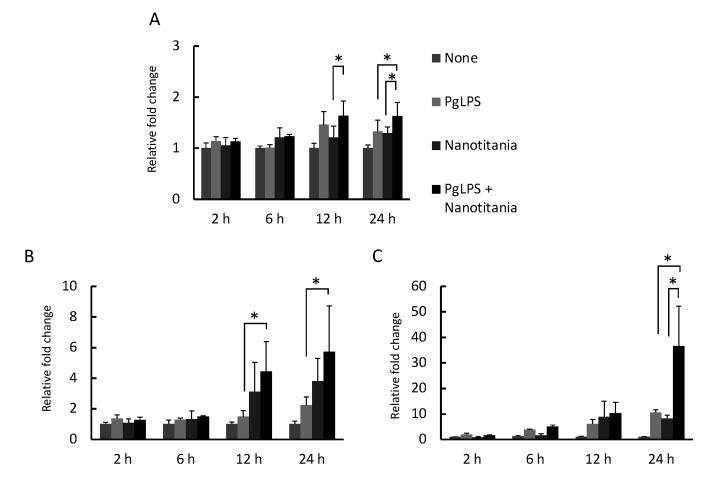
Expression levels of *IL-1β* (**A**), *IL-6* (**B**) and *IL-8* (**C**) mRNA were measured by qPCR after incubation with 10 µg/mL PgLPS, 10 µg/mL nanotitania and both stimulants together. Each data point indicates the average of six gene expression quantification results (n = 6). Bars indicate standard deviation, and asterisks indicate *p* < 0.05.

**Figure 5:**
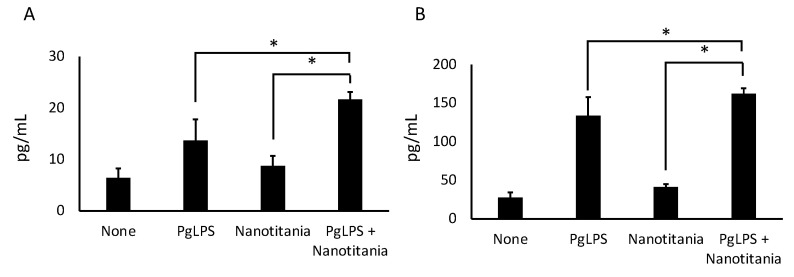
Protein levels of IL-6 (**A**) and IL-8 (**B**) were measured by ELISA after incubation with 10 µg/mL PgLPS, 10 µg/mL nanotitania and both stimulants together. Each data point indicates the average of six gene expression quantification results (n = 6). Bars indicate standard deviation, and asterisks indicate *p* < 0.05.

qPCR was performed to investigate whether inflammatory cytokine expression, which has been reported to be induced by PgLPS, is enhanced in the presence of nanotitania. *IL-1β* mRNA expression increased at 24 h by co-culture with PgLPS and nanotitania compared with PgLPS alone or nanotitania alone (Figure 4A). *IL-6* mRNA expression increased at 12 and 24 h by co-culture with PgLPS and nanotitania compared with PgLPS alone (Figure 4B). *IL-8* mRNA expression increased at 24 h by co-culture with PgLPS and nanotitania compared with PgLPS alone or nanotitania alone (Figure 4C). Additionally, IL-6 and IL-8 protein expression levels were significantly enhanced by co-culture with PgLPS and nanotitania compared with stimulation with PgLPS alone or nanotitania alone (Figure 5A,B).

### 2.5. Nanotitania Upregulates the Expression of IL-11

**Figure 6:**
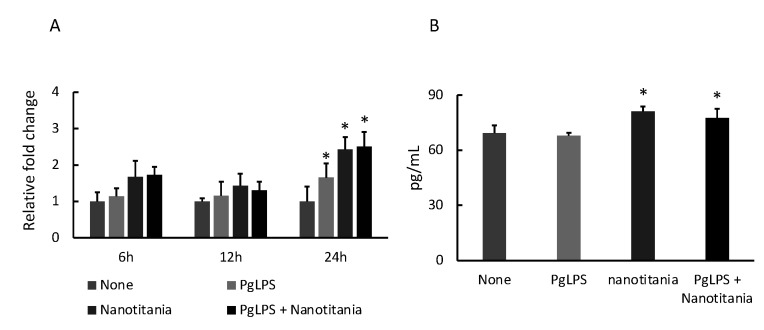
Expression levels of *IL-11* mRNA (**A**) and protein levels (**B**) of IL-11 were measured by qPCR or ELISA, respectively, after incubation with 10 µg/mL PgLPS, 10 µg/mL nanotitania and both stimulants together. Each data point indicates the average of six gene expression quantification results (n = 6). Bars indicate standard deviation, and asterisks indicate *p* < 0.05.

IL-11, involved in osteoclast differentiation, was examined by qPCR and ELISA. No significant difference was observed in *IL-11* mRNA expression when cultured with PgLPS or nanotitania alone or co-cultured with both reagents for 6 or 12 h. When stimulated with PgLPS or nanotitania alone or co-cultured with both reagents for 24 h, the expression of *IL-11* mRNA was significantly increased as compared with controls (Figure 6A). After 48 h incubation with nanotitania alone or co-cultured with PgLPS and nanotitania, IL-11 production was significantly higher than controls (Figure 6B).

## 3. Discussion

In the present study, the effects of nanotitania on gingival epithelial cells were investigated by qPCR, SEM observation and ELISA. A time- and dose-dependent effect due to the uptake of nanotitania was observed. At first, the expression of inflammatory cytokine (*IL-1β*, *IL-6* and *IL-8*) mRNA by stimulation with nanotitania alone was examined using qPCR. Expression of *IL-6* and *IL-8* mRNA in Ca9-22 was increased significantly after 12 h with 10 µg/mL nanotitania stimulation. On the other hand, when stimulated with 100 µg/mL nanotitania, both transcripts were significantly upregulated sooner from 2 h later.

As can be seen from the SEM images, it was speculated that the higher the concentration of nanotitania, the greater the amount of uptake into cells in a short period. It is unlikely that the large mass that can be seen in the SEM image is taken up into the cell all at once. However, it was observed that the higher the concentration of nanotitania, the faster it is taken up into the cell. Since nanotitania is not found in the nucleus of the cell, it may be stored in phagosomes in the cytoplasm. In fact, in cells cultured with 10 µg/mL of nanotitania, nanotitania was observed to accumulate intracellularly over time, and in cells cultured with 100 µg/mL of nanotitania, the nanotitania was observed as a large mass at 1 h incubation time. Since an increased mRNA expression of inflammatory cytokines was observed, the increase in nanotitania in cells may proportionally affect cytokine production. Therefore, to inhibit the uptake of nanotitania into the cells, dynasore was used to inhibit endocytosis [29]. The upregulated inflammatory cytokine mRNA by culture with nanotitania was significantly decreased by pre-treatment with dynasore. Therefore, the increase in inflammatory cytokine expression may be due to the uptake of nanotitania but not the effect of nanotitania on receptor-mediated signal transduction mechanisms.

Cellular uptake (endocytosis) mainly consists of phagocytosis and pinocytosis, but uptake of relatively small molecules is classified as pinocytosis and occurs in almost all cells [30]. The uptake of nanotitania in the present study is thought to be via pinocytosis because of the size of nanotitania particles and the type of cells investigated. This is consistent with the SEM image results. Pinocytosis is active in the epithelial cells of the small intestine and plays an important role in nutrient absorption [31]. However, the uptake of nanotitania by pinocytosis in the present study has a negative effect on the body and may contribute to the development and exacerbation of peri-implantitis. In addition, the enhancement of inflammatory cytokine expression by nanotitania was significantly increased by the presence of periodontopathic bacteria, regulated by the cellular signal transduction mechanism through the NF-κb pathway. This concomitant phenomenon may support the idea that intensified peri-implantitis progresses faster than chronic marginal periodontitis in actual clinical practice [7]. In particular, considering that the expression of IL-8 was significantly increased, inflammatory cells such as neutrophils gather due to the chemotaxis of IL-8, and the inflammation is exacerbated locally.

Furthermore, the expression of IL-11 was increased by the influence of nanotitania. IL-11, a member of the IL-6 family involved in pro-inflammatory and anti-inflammatory responses [32,33], is important for the maturation of megakaryocytes involved in hematopoiesis [34], but has also been reported to be involved in fat production [35], neurogenesis [36], hematopoiesis [37] and the formation of osteoclasts [38]. Since IL-11 acts as an osteolytic factor [39,40,41], the increased expression of IL-11 due to the influence of nanotitania may be a cause of the accelerated progression of peri-implantitis. Recently, there have been reports that overexpression of IL-11 is associated with a variety of cancers [42,43]. Therefore, increased expression of IL-11 due to the effects of nanotitania may be associated with exacerbation of peri-implantitis and possibly carcinogenesis.

This study is an in vitro study, and it is necessary to study these questions in vivo to see if the same effect is observed. In fact, in the clinical setting, there are contradictory data on the bacterial accumulation and inflammatory response when titanium or zirconia was used in implants [44]. In addition, the effects of nanotitania have only been investigated for up to 24 h, so it is necessary to consider a longer period. Overall, the results in this study show that uptake of nanotitania by gingival epithelial cells increases the expression of IL-6 and IL-8, which promotes inflammation, and that this effect is exacerbated by the presence of bacterial cell components of periodontopathic bacteria. In addition, increased IL-11 expression suggests that the effects of nanotitania are associated with alveolar bone destruction. Considering osteolysis due to wear debris in hip artificial joint prosthesis, macrophages phagocytize this wear debris, leading to the production of inflammatory cytokines and the maturation of osteoclasts [45,46,47]. In the present study, it was found that gingival epithelial cells phagocytize nanotitania, provoking the production of inflammatory cytokines in a similar fashion to that of macrophages facing micro-sized particles. In dental clinics, the presence and effects of nanotitania are not considered, and related studies are needed to further explore its effects. Therefore, the results of the present study show that the effects of titanium nanoparticles need to be considered when choosing titanium implants for dental treatment.

## 4. Materials and Methods

### 4.1. Cell Culture and Reagents

A human gingival epithelial cell line (Ca9-22), obtained from RIKEN BRC (Tsukuba, Japan), was cultured in Dulbecco’s modified Eagle’s medium (Life Technologies Japan, Tokyo, Japan) supplemented with 10% fetal bovine serum (Life Technologies Japan), 50 U/mL penicillin and 50 µg/mL streptomycin solution (Life Technologies Japan) at 37 °C in 5% CO_2_ atmospheric air. The cells from sub-confluent cultures were used after detachment with trypsin/EDTA (0.25%/1 mM). Titanium dioxide nano material (TTO-55 (A), nanotitania), with a size of <100 nm (mostly 30–50 nm), was obtained from Ishihara Sangyo Kaisha, Ltd. (Osaka, Japan). PgLPS was purchased from Wako (Osaka, Japan).

### 4.2. Sample Preparation for Reverse Transcription-Quantitative Polymerase Chain Reaction

Ca9-22 cells were seeded onto 24-well cell culture plates until they were 80% confluent (as pre-culture) for all experiments. To observe the effect of nanotitania, the medium was changed to fresh medium with 0.1% BSA containing nanotitania (10 or 100 µg/mL) and then incubated for 2, 4, 6, 12 and 24 h. For the nanotitania uptake inhibition assay, after pre-culture, the cells were pre-incubated with dynasore (10 or 50 µg/mL), which is an endocytosis inhibitor (Tokyo Chemical Industry Co., Ltd., Tokyo, Japan) for 30 min, then the medium was changed to fresh medium with 0.1% BSA containing nanotitania (10 or 100 µg/mL) and the samples were incubated again for 24 h. To examine the effects of nanotitania and PgLPS co-stimulation, the cells were stimulated with nanotitania (10 µg/mL) or PgLPS (10 µg/mL) or both reagents together for 2, 6, 12 and 24 h. After each incubation, total RNA was extracted from the cells using the RNeasy Mini Kit (QIAGEN, Copenhagen, Denmark) following the manufacturer’s instructions. Thereafter, cDNA was synthesized using the PrimeScript RT Master Mix (TaKaRa Bio, Shiga, Japan) and used as template sample for reverse transcription-quantitative polymerase chain reaction (qPCR).

### 4.3. Reverse Transcription-Quantitative Polymerase Chain Reaction

qPCR was performed as previously described [48]. Briefly, we used the StepOne Real-Time PCR system (Thermo Fisher Scientific, Waltham, MA, USA) following the manufacturer’s instructions. Primer sets for tumor necrosis factor α, *IL-1β*, *IL-6*, *IL-8*, *IL-11* and glyceraldehyde-3-phosphate dehydrogenase (*GAPDH*) were purchased from TaKaRa Bio (Shiga, Japan). *GAPDH* was utilized as an endogenous control. Cycle parameters were 95 °C for 30 s, followed by 40 cycles at 95 °C for 5 s and 60 °C for 30 s. Dissociation was performed each time to confirm the specificity of the primers.

### 4.4. Scanning Electron Microscope Observation

Scanning electron microscopy (SEM) evaluation was conducted as reported previously [49]. In brief, Ca9-22 cells were cultured in a cell culture insert at 37 °C in 5% CO_2_ atmospheric air for 48 h. Then, the cells were incubated with nanotitania (10 or 100 µg/mL) for 1, 12 and 24 h. After the incubation, cells on the culture insert were washed three times with phosphate-buffered saline (PBS; Nacalai Tesque, Kyoto, Japan) and fixed with 2.5% glutaraldehyde for 1 h. After washing with PBS, the cells were post-fixed with 1% OsO_4_ at 4 °C for 1 h. The cells were washed again three times with PBS and then dehydrated with 50%, 70%, 80% and 90% ethanol for 15 min each, and then the cells were washed with 100% ethanol four times for 15 min. After treating with QY-1 (Nissin EM, Japan), the cells on the membrane were incubated in a 1:1 mixture of epoxy resin (Epon 812 TAAB Laboratories, Aldermaston, Berks, UK) and QY-1 overnight. The cells on the membrane were then embedded in fresh epoxy resin at a room temperature of 25 °C (RT) for 6 h and then at 60 °C for 48 h. The samples were cut into 100 nm thin sections and mounted on a glass slide, which was coated with platinum to make the surface conductive. The samples containing cells with or without nanotitania were stained with 1% uranyl acetate solution at RT for 30 min and then were stained with lead nitrate solution at RT for 5 min. Back-Scattered electron images of the surface of the samples were observed using a SU8010 SEM (Hitachi High-Technologies, Tokyo, Japan).

### 4.5. Enzyme-Linked Immunosorbent Assay

The culture supernatants were utilized for enzyme-linked immunosorbent assay (ELISA). Ca9-22 cells were pre-cultured in the same conditions as for qPCR. The culture supernatants of 24 h (IL-6 and IL-8) or 48 h (IL-11) cultured with or without reagents were collected. The concentration of inflammatory cytokines (IL-6 and IL-8) and IL-11 in the culture supernatant was measured using an ELISA kit (IL-6 and IL-8: R&D Systems, MN, USA; IL-11: Abcam, Cambridge, UK) according to the manufacturer’s instructions.

### 4.6. Statistical Analysis

In the graphs, all values are shown as mean ± standard deviation. MacTKV3 software (Esumi Co., Ltd., Tokyo, Japan) was used for statistical analysis. Analysis of variance with Bonferroni’s post-test was used, and statistical significance was indicated as * *p* < 0.05. Normality was confirmed by the Shapiro–Wilk test for all sample groups before parametric tests.
